# Global Proteomic Analysis of Mesenchymal Stem Cells Derived from Human Embryonic Stem Cells via Connective Tissue Growth Factor Treatment under Chemically Defined Feeder-Free Culture Conditions

**DOI:** 10.4014/jmb.2110.10032

**Published:** 2021-11-06

**Authors:** Ji-Hye Seo, Young-Joo Jeon

**Affiliations:** 1 Department of Dental Pharmacology, School of Dentistry, Jeonbuk National University, Jeonju 54896, Republic of Korea; 2 Stem Cell Convergence Research Center, Korea Research Institute of Bioscience and Biotechnology (KRIBB), Daejeon 34141, Republic of Korea

**Keywords:** Mesenchymal stem cell, CTGF, proteomics, Wnt/β-catenin, cell therapy

## Abstract

Stem cells can be applied usefully in basic research and clinical field due to their differentiation and self-renewal capacity. The aim of this study was to establish an effective novel therapeutic cellular source and create its molecular expression profile map to elucidate the possible therapeutic mechanism and signaling pathway. We successfully obtained a mesenchymal stem cell population from human embryonic stem cells (hESCs) cultured on chemically defined feeder-free conditions and treated with connective tissue growth factor (CTGF) and performed the expressive proteomic approach to elucidate the molecular basis. We further selected 12 differentially expressed proteins in CTGF-induced hESC-derived mesenchymal stem cells (C-hESC-MSCs), which were found to be involved in the metabolic process, immune response, cell signaling, and cell proliferation, as compared to bone marrow derived-MSCs(BM-MSCs). Moreover, these up-regulated proteins were potentially related to the Wnt/β-catenin pathway. These results suggest that C-hESC-MSCs are a highly proliferative cell population, which can interact with the Wnt/β-catenin signaling pathway; thus, due to the upregulated cell survival ability or downregulated apoptosis effects of C-hESC-MSCs, these can be used as an unlimited cellular source in the cell therapy field for a higher therapeutic potential. Overall, the study provided valuable insights into the molecular functioning of hESC derivatives as a valuable cellular source.

## Introduction

Recently, research has focused on several kinds of stem cells and their possible clinical applications in cell replacement therapy due to their specific abilities, including self-renewal and differentiation into different kinds of lineages. Two major types of stem cells, embryonic stem cells (ESCs), which can be isolated from the inner cell mass of the blastocyst of embryo, and adult stem cells, which can be obtained from the bone marrow, cord blood, adipose tissue, and so on, have been widely used. However, ESCs, including human embryonic stem cells (hESCs), have ethical and legal issues, and several kinds of adult stem cells, including mesenchymal stem cells (MSCs), are also limited by the cellular source. Thus, in this study, we aimed to establish an MSC population from hESCs via treatment with connective tissue growth factor (CTGF) under chemically defined feeder-free culture conditions [1-3].

MSCs are a promising cellular source for tissue engineering and regenerative medicine. They are stromal cells derived from a variety of adult tissue sources such as umbilical cord, endometrial polyp, menstrual blood, bone marrow, adipose tissue, and so on. Embryonic stem cells possess the ability for self-renewal and exhibit multilineage differentiation into the mesodermal lineage such as in osteogenesis, adipogenesis, and chondrogenesis. Moreover, ESCs can produce neurons, endothelial cells, Schwann cells, medullary nucleus cells, cardiomyocytes, and alveolar epithelial cells [4], and ESCs are recognized by the expression of unique cell surface markers, such as a positive expression for the cluster of differentiation (CD) 29, CD44, CD105, CD73, CD90, and CD166 and a negative expression for the hematopoietic markers CD45, CD34, CD14 or CD11b, CD79α or CD19, and major histocompatibility complex (MHC) II cell surface receptor (HLA-DR) [5].

Prior to the clinical application of MSCs, major challenges remain such as the elucidation of the highly complex underlying mechanisms, including the related mechanisms for differentiation, mobilization, and homing of MSCs, in vitro and in vivo. Given these, we aimed to perform a proteomic analysis on CTGF-induced hESC-MSCs to understand the molecular expression profile governing the therapeutic mechanism of MSCs and to discover the optimal cell type for clinical use, on the basis of the two-dimensional map.

The fundamental differences at the molecular level between spontaneously differentiated hESC-MSCs (hESC-MSCs) and chemically differentiated hESC-MSCs treated with CTGF (C-hESC-MSCs) when compared with bone marrow-derived MSCs (BM-MSCs) are largely unknown. Thus, in this study, to elucidate the difference between these MSCs at the protein level, we applied two-dimensional gel electrophoresis (2-DE) to the proteomic analyses to build a comprehensive database of the stem cell proteome, followed by liquid chromatography–tandem mass spectrometry (LC–MS/MS) to investigate differentially expressed proteins between hESC-MSCs, C-hESC-MSCs, and BM-MSCs. Twelve proteins were identified to be upregulated in the C-hESC-MSCs, which are involved in several different biological processes, including metabolic process, immune response, cell signaling, and cell proliferation.

Here, we treated CTGF, also known as cellular communication network (CCN) factor 2 (CCN2), on feeder-free cultured naive hESCs to chemically induce mesenchymal differentiation [6]. Connective tissue growth factor belongs to the CCN family of extracellular-matrix-associated heparin-binding proteins and is also secreted as a multifunctional signaling modulator in various biologic or pathologic processes, including cell adhesion, migration, growth, chemotaxis, and in the synthesis and accumulation of extracellular matrix (ECM) during pathologic conditions. Moreover, CTGF plays a crucial role in tissue remodeling during inflammation and fibrosis. Cellular communication network families have also been reported to show multiple interactions between them and with other growth factors such as transforming growth factor β (TGF-β), bone morphogenetic protein (BMP), and insulin-like growth factor, and the interactions between these growth factors are made possible by the ECM, cell surface receptors such as integrins, and especially the multimodular character of the CCN factors [7-9].

In a study, CTGF was reported to act either as an oncoprotein or a tumor suppressor among different cancers, wherein CTGF enhanced the stem-like properties and increased the expression of multiple pluripotency genes in head and neck squamous cell carcinoma [10]. In in vivo studies, CCN2-deficient mice have been shown to die soon after birth as a result of severe skeletal abnormalities associated with impaired chondrocyte proliferation and ECM production. These in vivo findings support the in vitro finding that CCN2 is a promoter of cell proliferation and differentiation during endochondral ossification [11]. In mechanistic studies related with CTGF, it has been reported that CTGF induces c-Jun expression through the αvβ3-integrin and that c-Jun directly activates the transcription of the pluripotency genes *NANOG*, *SOX2*, and *POU5F1* [12]. Also, CTGF has been reported to play an important role in the pathogenesis of epithelial–mesenchymal transition (EMT) via activation of the canonical Wnt signaling in renal tubular epithelial cells and to induce the transdifferentiation of normal tubular epithelial cells into mesenchymal cells via activation of the Wnt/β-catenin signaling pathway in human kidney 2 (HK-2) cells in vitro [13].

Similar to these previous studies, the activation of Wnt signaling by CTGF has been reported in mesangial cells and *Xenopus laevis* embryos by the stimulation of lipoprotein receptor-related proteins 5 and 6 (LRP5/6), LRP6, and glycogen synthase kinase-3β (GSK-3 β), resulting in the accumulation and nuclear localization of β-catenin, TCF/LEF activity, and expression of Wnt targets. In a study on nucleus pulposus cells, it was shown that Wnt–β-catenin signaling regulates the expression of CTGF through the mitogen-activated protein kinase (MAPK) pathway [14]. Over the past several decades, Wnt signaling has been identified to play an essential role in cell fate determination, proliferation, and differentiation. Dysregulation/hyperactivation of Wnt signaling is associated with numerous diseases such as neurodegeneration, gastrointestinal cancers, and osteoporosis, and, ultimately, β-catenin-dependent Wnt signaling elicits gene transcriptional activity to influence MSC lineage determination, and Wnt proteins might play an important role in governing the MSC cell fate [15-17] .

In this study, we aimed to obtain a C-hESC-MSCs cell population under chemically defined differentiation conditions and analyze specific proteome expression profile using a 2-DE- and LC–MS/MS-based proteomic analysis tool. As the result, we were able to propose candidate molecules that were specifically upregulated in the C-hESC-MSCs, including X-ray repair cross complementing 5 (XRCC5), insulin-like growth factor 2 mRNA-binding protein 1 (IGF2BP1), histone binding protein 4 RBBP4 (RBBP4), nucleophosmin 1 (NPM1), proliferating cell nuclear antigen (PCNA), guanine nucleotide-binding protein G(i), alpha-2 subunit (GNAI2), prohibitin 2 (PHB2), laminin subunit beta-1 precursor (LAMB1), laminin subunit gamma-1 precursor (LAMC1), annexin A3 (ANXA3), proteosome activator complex subunit 3 (PSME3), and proteosome 26S subunit, non-ATPase 5 (PSMD5), and these are involved in the metabolic process, immune response, cell signaling, and cell proliferation and probably the most related to the Wnt/beta-catenin pathways. The discovery of the molecular targets associated with CTGF and the Wnt signaling pathway provide a valuable contribution to the field of cell therapy by offering not only a valuable novel cellular source but also elucidating their therapeutic mechanism.

## Materials and Methods

### Culture of the hESCs

The undifferentiated hESCs (H9 hESC) were cultured on mitotically inactivated ouabain-resistant (STO) feeder cells (ATCC, USA) in Dulbecco’s Modified Eagle Medium/Nutrient Mixture F-12 (DMEM/F12; 50: 50; Gibco BRL, USA) supplemented with 20% (v/v) serum replacement (Gibco, USA) containing basic embryonic stem (ES) medium components, including 1 mM L-glutamine (Gibco), 1% nonessential amino acids (Gibco), 100 mM beta mercaptoethanol (Gibco), and 4 ng/ml bFGF (Sigma, USA). Every 5 or 6 d, the hESCs were detached with a dissecting pipette and transferred to new cell culture dishes with mitomycin C-treated STO feeder cells, as previously described [2]. Our research was performed under ethical approval from the Institutional Review Board (IRB) at Korea Research Institute of Bioscience and Biotechnology.

### Induction of MSC Differentiation from Naïve hESCs and Their Cultivation

To induce differentiation of the naïve-hESCs into mesenchymal lineage cells, we first adapted the hESCs on a feeder-free culture condition using the mTeSR medium kit (STEMCELL Technologies Inc., Canada) on Matrigel hESC-qualified Matrix (BD Biosciences) with 20% (v/v) serum replacement (Gibco) for 5 d to verify the xeno-free differentiation condition. Then, the cells were differentiated into the MSC cell population via treatment with 100 ng/ml of CTGF for an additional 5 d in same cell culture medium. Finally, the differentiated cells were maturated into the MSC population in EBM cell culture medium (Lonza, USA) treated with 25 ng/ml of CTGF. The EBM medium consists of ascorbic acid, GA-1000, hydrocortisone, and 2% SR. After the final differentiation, the CTGF-induced hESC-MSCs were cultured in EMB medium and expanded through the typical trypsinization method.

### Spontaneous Differentiation of hESCs into MSCs

To induce spontaneous differentiation of hESCs into MSCs, the feeder-free cultured hESCs for 5~6 d were transferred into a new cell culture medium consisting of EBM and 2% fetal bovine serum (FBS), without serum replacement for 4 d, according to our previous study [18].

### Culture of BM-MSCs

The BM-MSCs were purchased from Lonza, and cell culture was performed as per the manufacturer’s instruction in the MSCGM cell culture medium kit (Lonza).

### Flow cytometry

For the fluorescence-activated cell sorting (FACS) analysis, the cells were dissociated with 0.25% trypsin–ethylenediaminetetraacetic acid (EDTA; Gibco) and washed with phosphate-buffered saline (PBS) containing 2%(v/v) FBS. The cells were incubated with either the isotype control or antigen-specific antibodies for 20 min: CD44, CD90, CD73, CD105, CD34, and CD45 (R&D systems, USA). Propidium iodide staining (5 ng/ml) was used to identify the nonviable cells. The FACS analysis was performed using a FACS Caliber FlowCytometer (BD Bioscience) and analyzed using the CellQuest Pro (BD Bioscience).

### Two-Dimensional Electrophoresis Analysis

We compared the protein expression profiles of the BM-MSCs, hESC-MSCs, and C-hESC-MSCs using 2-DE. Pro-Prep Protein Extraction Solution (iNtRON Biotechnology, Korea) was added to each sample, and the total protein (300 ug) for the analytical runs was transferred into IPG strip holder channels (Amersham Biosciences, USA). The 2-DE protein mixtures were separated by isoelectric focusing (IEF) in the first dimension and sodium dodecyl sulfate (SDS)-polyacrylamide gel electrophoresis (PAGE) in the second dimension. The total proteins were mixed with a rehydration solution (9 M urea, 2 M thiourea, 4% [w/v]CHAPS, 50 M DTT, and a trace of bromophenol blue) for a final volume of 300 ml, and then incubated for 12 h at room temperature (RT) before separation by IEF at 250 V for 15 min, 1,000 V for 2 h, or 1,000 V for 6 h, with 50 mA per gel strip. The gel strips were then immediately equilibrated in equilibrium buffer (50 mM Tris-HCl, pH 8.8, 6 M urea, 30% [v/v] glycerol, and 2% [w/v] SDS). After equilibration, the IPG gel strips were transferred for the second dimension onto the SDS-PAGE, followed by electrophoresis in a Protean II xi 2-DE cell (Bio-Rad) at 20 mA. The 2-DE gels were visualized using a Silver Staining Kit according to manufacturer’s protocol (GE Healthcare).

### Identification of Proteins by LC–MS/MS

The resulting tryptic peptides were separated and analyzed using a Waters Nano LC system equipped with a Waters C18 nano column (75 um × 15 cm, nanoAcquityTM UPLCTM column). The peptides were eluted from the column with a gradient ranging from 2% to 40% of binary solvent B1 for 30 min at 0.4 ml/min. The lock mass,[Glu1] fibrinopeptide at 400 fmol/ml, was delivered from the auxiliary pump of the Nano LC system at 0.3 ml/min to the reference sprayer of the NanoLockSprayTM source. Mass spectrometry analysis of the tryptic peptides was performed using a Waters SynaptTM HDMS. The mass spectrometer was operated in V-mode for all measurements. All analyses were performed using a positive mode Nano ESI with a NanoSpray source. The lock mass channel was sampled every 30 s. The mass spectrometer was calibrated using a [Glu1] fibrinopeptide solution (400 fmol/ml) delivered through the reference sprayer of the NanoLockSpray source. Accurate mass LC–MS/MS data were collected via the data-dependent acquisition mode of acquisition. The processed ions were sequenced and mapped against the Swiss-Prot database using the MASCOT DAEMON programs (http://www.matrixscience.com). The peptides were restricted to the trypsin fragments, with a maximum of one missed cleavage, and cysteine carbamidomethylation and identification of the protein in at least two out of three replicate injections were performed.

### Bioinformatic and Data Analyses

Functional annotation clustering and the Kyoto Encyclopedia of Genes and Genome (KEGG) pathway analysis was performed using the DAVID (Database for Annotation, Visualization and Integrated Discovery, version 6.8) (http://david-d.ncifcrf.gov). Heatmap with clustering analysis was visualized using the MeV 4.9.0 software with the K-means method and Euclidean Distance.

### Western Blotting

The cells were washed with ice-cold PBS and lysed with M-PER Mammalian Protein Extraction Reagent (Thermo Scientific, USA) containing a protease inhibitor. Equal amounts of 20~40 ug of total protein were separated on 10% or 15% v/v SDS–PAGE, and then transferred onto polyvinylidene difluoride (PVDF) membranes (Millipore, USA). The membrane was blocked with 5% non-fat dried milk in Tris-buffered saline with 0.1% Tween 20 detergent (TBS–T), and then incubated with the primary antibody overnight on a rocking platform at 4°C. The membrane was then washed four times with TBS–T buffer for 10 min and incubated with 5%skim milk in TBS–T buffer containing horseradish peroxidase-conjugated secondary antibody for 1 h. The hybridized membrane was washed in TBS–T buffer, and specific bands were detected using a chemiluminescent ECL detection kit (GE Healthcare).

### Antibodies

Antibodies against octamer-binding transcription factor 4 (1:1000, Oct-4; ab200834, abcam), Brachyury (1:1000, sc-166962, Santa Cruz Biotechnology), GATA-binding protein 4 (1:1000, GATA4; sc-25310, Santa Cruz Biotechnology), SRY box transcription factor 17 (1:1000, Sox17; AF1924, R&D system), neuronal differentiation 1 (1:1000, NeuroD1; ab205300, abcam), XRCC5 (1:2000, AF5819, R&D system), IGF2BP1 (1:1000, ab184305, abcam), RBBP4 (1:1000, MAB7416, R&D system), NPM1 (1:1000, sc-271737, Santa Cruz Biotechnology), PCNA (1:1000, sc-56, Santa Cruz Biotechnology), GNAI2 (1:1000, ab137050, abcam), PHB2 (1:1000, sc-133084, Santa Cruz Biotechnology), LAMB1 (1:1000, sc-17810, Santa Cruz Biotechnology), LAMC1 (1:1000, sc-17751, Santa Cruz Biotechnology), β-catenin (1:1000, sc-7963, Santa Cruz Biotechnology), AKT (1:2000, #4691, Cell Signaling Technology), Jun amino-terminal kinases (JNK) (1:1000, #9252, Cell Signaling Technology), phosphorylated (p)-JNK (1:1000, #9255, Cell Signaling Technology), p38 (1:1000, sc-81621, Santa Cruz Biotechnology), p-p38 (1:1000, #9216, Cell Signaling Technology), TGF-β (1:1000, #3711, Cell Signaling Technology), CTGF (1:1000, sc-385970, Santa Cruz Biotechnology), and glyceraldehyde 3-phosphate dehydrogenase (GAPDH; (1:3000, sc-47724, Santa Cruz Biotechnology) were employed for the western blot analysis.

### Statistical Analysis

Quantitative data were expressed as the mean ± standard deviation (SD). Statistical analysis was performed using the *t*-test. A value of *p* < 0.05 was considered statistically significant. The significance of the differences was evaluated by paired two-tailed student’s *t*-tests (SAS version 8.0, USA).

## Results

### Differentiation of hESCs into MSC via CTGF Treatment under a Chemically Defined Condition

In this study, to induce the differentiation of the H9 hESCs into mesenchymal lineage cells, we conducted a two-step CTGF treatment method on naïve hESCs, which were cultured under a chemically defined feeder-free culture condition.

**Fig. 1:**
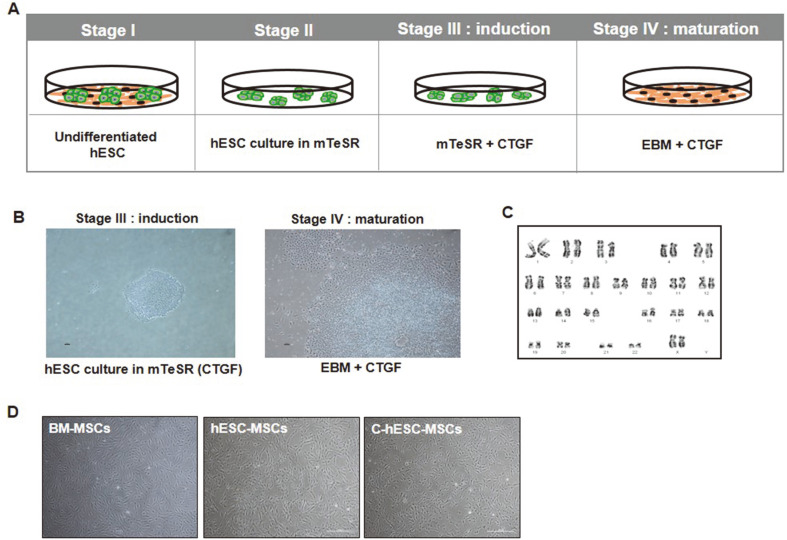
Experimental scheme and differentiation of the hESCs into MSCs via CTGF treatment. (**A**) Experimental scheme for the differentiation of the hESCs into MSCs via CTGF treatment. (**B**) Morphological analysis during the differentiation of the hESCs into MSCs. Feeder-free culture of the hESCs in mTeSR medium on Matrigel hESC-qualified matrix-coated cell culture dishes for 5 d with 100 ng/ml of CTGF treatment in the induction stage (left panel, 10X magnification) and C-hESC-MSCs on the maturation stage (right panel, 10X magnification) with 25 ng/ml of CTGF treatment. (**C**) Karyotype (44 + XY) analysis of the C-hESC-MSCs at passage 3. (**D**) Phase-contrast images of the BM-MSCs, hESC-MSCs, and C-hESC-MSCs after three passages (40X magnification).

In Fig. 1A, we describe the experimental scheme used to induce differentiation of the hESCs into MSCs via a two-step CTGF treatment. H9 hESCs were transferred to a feeder-free culture condition, consisting of the mTeSR culture medium coated with Cell Start on the culture dish, to induce a chemically defined condition during differentiation. The H9 hESCs under the feeder-free condition were cultured for an additional 5–7 d. Then, to induce differentiation of the hESCs into MSCs (first induction step with CTGF) via the CTGF treatment, we treated the cells with 100 ng/ml of CTGF for 5 d (Fig. 1B, left panel) in the mTeSR culture medium in Cell Start-coated plates, and for the second differentiation maturation step, 25 ng/ml of CTGF for 5 d was added (Fig. 1B, right panel) in the EBM cell culture medium condition. During differentiation, we also performed karyotype analysis to confirm genetic stability during the differentiation period. As a result, we confirmed that genetic stability was sustained during the differentiation of the C-hESC-MSCs from the H9 hESCs (Fig. 1C). At confluence, the C-hESC-MSCs exhibited a typical mesenchymal fibroblast-like morphology (Fig. 1D, right panel), and the cultivation and expansion of the cells were continued using the trypsinization method in the same cell culture condition.

Three types of MSCs (BM-MSCs, spontaneously differentiated hESC-MSC [hESC-MSCs], and C-hESC-MSC) obtained from different cellular sources and differentiation methods retained similar phenotypic characteristics. After the differentiation and maturation of the three types of MSCs, all types of MSCs could expand using the typical trypsinization method, and the proliferating MSCs exhibited fibroblast- and spindle-like morphology after passaging (Fig. 1D). The C-hESC-MSCs exhibited a higher proliferative potential than those of the other control cells such as the hESC-MSCs or BM-MSCs. The C-hESC-MSCs could be cultured up to the 10^th^ passage, and we used three passage cells in all the proteomic experiments (data not shown).

### Phenotypic Characterization of C-hESC-MSCs

Then, to confirm the specific marker expression profile of the C-hESC-MSCs as MSCs, comparing with the BM-MSCs and hESC-MSCs, we performed western blot and FACs analyses at passage 3. We compared the general undifferentiation and mesodermal differentiation marker expression profiles of the BM-MSCs, hESC-MSCs and C-hESC-MSCs.

**Fig. 2:**
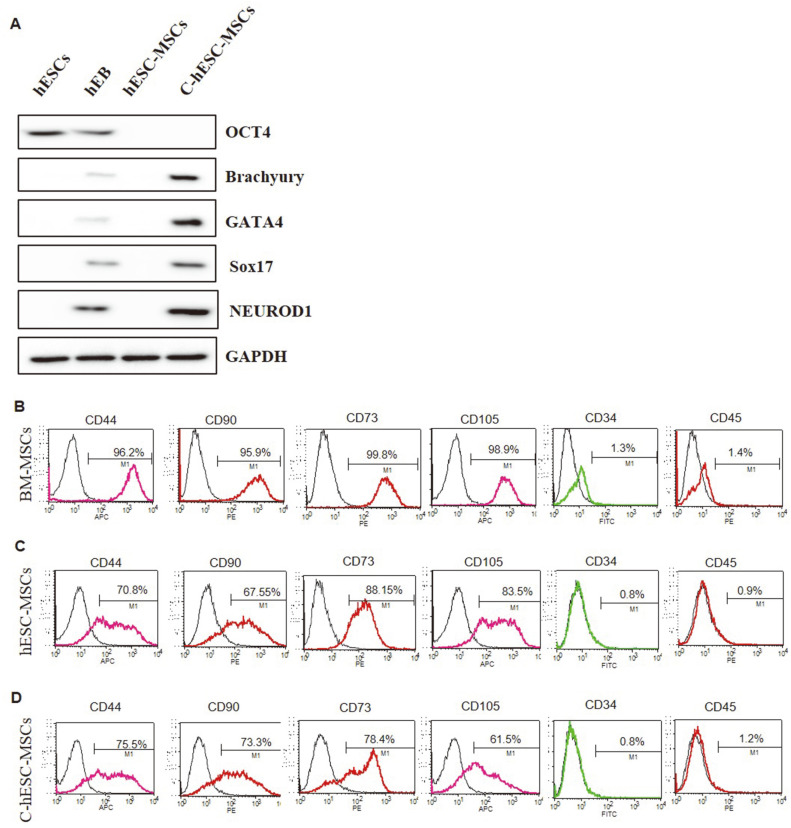
Phenotypic characterization of the BM-MSCs, hESC-MSCs, and C-hESC-MSCs. (**A**) Western blotting analysis of the undifferentiation and differentiation markers, including octamer-binding transcription factor 4 (Oct-4; undifferentiation), Brachyury (mesodermal), GATA-binding protein 4/SRY box transcription factor 17 (GATA 4/Sox17; endodermal), and neuronal differentiation 1 (NeuroD1; ectodermal) in the BM-MSCs, hESC-MSCs, and C-hESC-MSCs. Day 10 human embryoid bodies (hEBs) were used as the differentiation control. (**B, C**, and **D**) Fluorescence-activated cell sorting (FACS) analysis of the BM-MSCs, hESC-MSCs, and C-hESC-MSCs for MSC surface markers, including cluster of differentiation (CD) CD44 (adhesion marker), CD90 (mesenchymal marker), CD73 (adhesion marker), and CD105 (mesenchymal marker) and confirmation of the negative expression of hematopoietic cell surface markers such as CD34 and CD45.

In the western blotting analysis, we checked the expression of the undifferentiation (Oct-4), and the mesodermal (Brachyury), endodermal (GATA 4 and Sox17), and ectodermal (NeuroD1) differentiation markers in the naïve hESCs and in the three types of MSCs. We used day 10 human embryoid bodies (hEBs) as the differentiation control. The hESC colonies were detached from the feeder layers using a dissecting pipette and spontaneously formed hEBs in a suspension culture. As shown in Fig. 2A, the expression of Oct4 was dramatically reduced during differentiation. Genes encoding the mesodermal differentiation factor, including Brachyury, GATA 4, and Sox17, were all upregulated in the differentiated C-hESC-MSCs relative to the hEBs, while the hESC-MSCs showed an extremely low expression of Brachyury, GATA 4, and Sox17 (Fig. 2A).

The MSC phenotype of the expanded C- hESC-MSCs was characterized by measuring the positive expression of mesenchymal stem cell surface marker such as CD44 (adhesion marker), CD90 (mesenchymal marker), CD73(adhesion marker) and CD105 (mesenchymal marker), and negative expression of hematopoietic cell surface marker such as CD34 and CD45 (Figs. 2B-2D). C-hESC-MSCs were extremely positive for CD44 (over 75%), CD90 (over 73%), CD73 (over 78%) and CD105 (over 61%), whereas CD34 and CD45 were expressed negatively in all paired samples. As the result, we confirmed that the differentiated C-hESC-MSCs retained their mesenchymal stem cell characteristics and are similar to purchased BM-MSCs.

### Global Proteomic Analysis of the BM-MSCs, hESC-MSCs, and C-hESC-MSCs

**Fig. 3:**
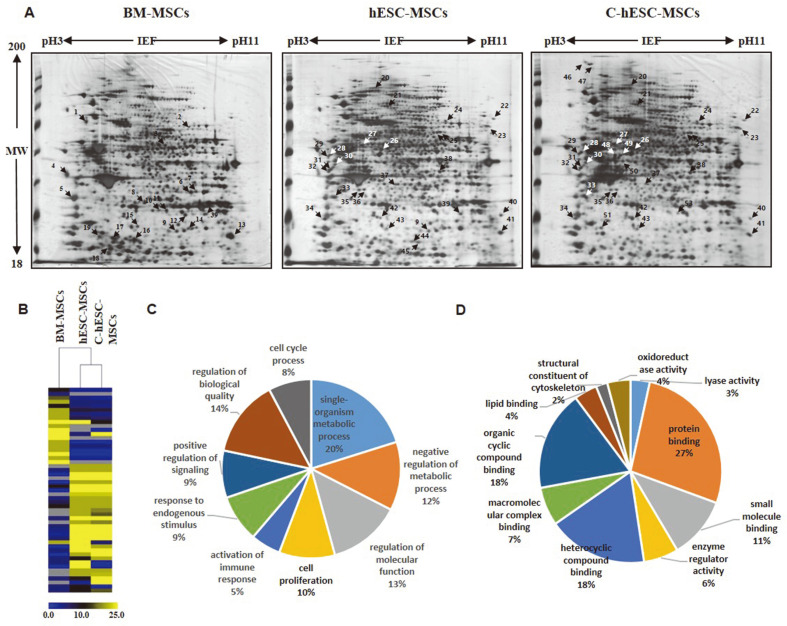
Global proteomic analysis of the BM-MSCs, hESC-MSCs, and C-hESC-MSCs. (**A**) Two-dimensional (2D) gel electrophoresis (2-DE) images for the proteins isolated from the BM-MScs (left panel), hESC-MSCs (middle panel), and C-hESC-MSCs (right panel). Among the 3,000 spots, 61 proteins were significantly up- or downregulated, respectively, by intensities of at least 1.5-fold in the C-hESC-MSCs relative to those of the BM-MSCs. (**B**) Heat map of cluster analysis for the proteins based on the 2-DE proteomic and LC-MS/MS protein identification results. (**C** and **D**) Ontological classification of the differentially regulated proteins in terms of the related biological process and molecular function using the Gene Ontology (http://www.geneontology.org) and UniProt (http://www.expasy.uniprot.org) websites.

To further elucidate the comparative proteomic profiling map of each MSCs, we applied powerful tool, a 2-DE-based proteomic approach, for discovering the regulatory networks at the molecular level. Three types of MSCs, including BM-MSCs, hESC-MSCs, and C-hESC-MSCs, were harvested at passage 3, and as the first step in this 2-DE proteomic approach coupled with LC–MS/MS protein identification, we created a reference master gel. Three independent replications of the experiment were performed to certify the reproducibility of the protein homogenates on the 2-DE gels. Then, the analytical gels were subjected to silver staining for visualization (Fig. 3A). In Fig. 3A, results of 2-DE gel image for BM-MSCs and hESC-MSCs (right and middle panel in Fig. 3A) were quote from our previous study [18]. Among the 3,000 spots, 61 proteins were significantly up- or downregulated, respectively, by intensities of at least 1.5-fold in the hESC-MSCs and C-hESC-MSCs, respectively, relative to the BM-MSCs, and these proteins are listed in Table 1. Differentially expressed protein spots were assigned a unique number, wherein black and white arrows were used to identify the spots during the gel-matching process. A hierarchical cluster analysis was performed in the BM-MSCs, hESC-MSCs, and C-hESC-MSCs with differentially expressed proteins. The proteins of the hESC-MSCs and C-hESC-MSCs were very similar to those of the BM-MSCs, and some of them were expressed differently (Fig. 3B).

The list of identified proteins was then classified according to the related biological processes and molecular function on the basis of their functional involvement using information from the DAVID database (https://david.ncifcrf.gov/summary.jsp) and UniProt (http://www.expasy.uniprot.org) websites; the results were displayed as a graph (Figs. 3C and 3D). A database search and functional exploration of these differentially expressed proteins showed that these proteins have different roles that can be related to the metabolic process, immune response, cell signaling, and cell proliferation, on the basis of their functions.

A database search and functional exploration of these differentially expressed proteins revealed that these proteins have different roles according to their biological processes (Fig. 3C), which are divided into nine categories, namely, single-organism metabolic process (20%), negative regulation of metabolic process (12%), regulation of molecular function (13%), cell proliferation (10%), activation of immune response (5%), response to endogenous stimulus (9%), positive regulation of signaling (9%), regulation of biological quality (14%), and cell cycle proves (8%). Also, according to their molecular function (Fig. 3D), the differentially expressed proteins were dividede into 10 categories, namely, oxidoreductase activity (4%), lyase activity (3%), protein binding (27%), small molecule binding (11%), enzyme regulator activity (6%), heterocyclic compound binding (18%), macromolecular complex binding (7%), organic cyclic compound binding (18%), lipid binding (4%), and structural constituent of cytoskeleton (2%).

### Validation of the Differentially Expressed Proteins in the C-hESC-MSCs, Which are Involved in Cell Proliferation, by Immunoassay

By correlating the nine functional categories with the biological processes analysis, the identified proteins were shown to have characteristics such as cell proliferation, differentiation, innate immune response, transcriptional regulation, mammalian embryogenesis, cell cycle arrest, DNA repair, apoptosis, cell survival, tumorigenesis, invasion, and metastasis, and these related functions may play an important role for the maintenance of stem cell activities. After analyzing the identified proteins, in this study, we focused on the functionally clustered proteins that are involved in the cell proliferation due to their important roles and capacity in cell differentiation and cell engraftment/homing in vivo after cell transplantation.

**Fig. 4:**
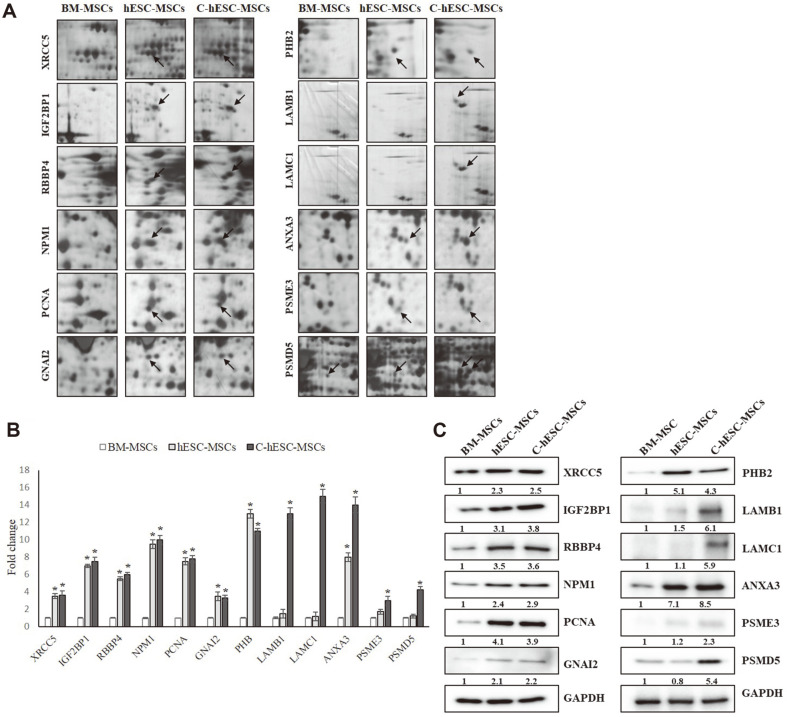
Verification of the upregulated expression pattern of the differentially expressed proteins in the ChESC- MSCs using western blotting. (**A**) Magnified images of the differentially expressed proteins. The arrows indicate the location of each protein feature. (**B**) Quantification of differentially expressed protein spots. Intensities of each spot were analyzed and presented in a histogram. Results are means ± S.E.M for at least three independent experiments. The significance of differences was evaluated by paired two-tailed Student’s t test. *p < 0.05 compared with BM-MSCs. (**C**) Western blot analysis of the BM-MSCs, hESC-MSCs, and C-hESC-MSCs (lane 1, BM-MSCs; lane 2, hESC-MSCs; lane 3, C-hESC-MSCs) using specific antibodies that are recognized by the proteins identified through 2-DE. Glyceraldehyde 3-phosphate dehydrogenase (GAPDH) was used as the loading control.

Magnified comparison maps of these representative protein spots in the 2-DE maps for the hESC-MSCs and C-hESC-MSCs relative to the BM-MSCs are visualized separately in Fig. 4A. The expression quantification of each specific protein is presented in Fig. 4B as a grouped bar chart with error bars. Each bar represents the volume means ± SD of gels from three independent experiments. When correlated with their biological process, proteins involved in cell proliferation, including XRCC5, IGF2BP1, RBBP4, PCNA, NPM1, GNAI2, PHB2, LAMB1, LAMC1, ANXA3, PSME3 and PSMD5, were specifically up-regulated in the CTGF-induced hESC-MSCs relative to the BM-MSCs; thus, these proteins were selected for use in validating the expression pattern using western blot techniques. As shown in Fig. 4C, immunoblotting with antibodies specific to XRCC5, IGF2BP1, RBBP4, NPM1, PCNA, GNAI2, PHB2, LAMB1, LAMC1, ANXA3, PSME3, and PSMD5 were used to examine the protein expression patterns in the three types of MSCs.

As shown in Fig. 4B, similar to that of the 2-DE analysis, the 12 specifically up-regulated proteins also retained up-regulated expression patterns in the C-hESC-MSCs as compared to those of the hESC-MSCs and BM-MSCs.The XRCC5, IGF2BP1, and RBBP4 were upregulated in the C-hESC-MSCs at about 2.5-, 3.8-, and 3.5-fold changes, respectively, when compared with those of the BM-MSCs. The upregulated expression patterns of NPM1, PCNA, GNAI2, and PHB2 in the C-hESC-MSCs were shown to have 2.9-, 3.9-, 2.2-, and 4.3-fold changes, respectively, and 6.1-, 5.9-, 8.5-, 2.3-, and 5.4-fold changes were verified in the LAMB1, LAMC1, ANXA3, PSME3, and PSMD5 (Fig. 4B) when compared with those of the BM-MSCs. The subset contained proteins with a variety of biological functions, suggesting that a relatively wide range of proteins were represented.

### Understanding the Relationships between the Cell Signaling Pathway Related with Cell Survival, Apoptosis, and Differentiation and the Upregulated Proteins

To confirm whether the CTGF treatment induced hESC differentiation into MSCs and can modulate the Wnt–β-catenin signaling, cell survival/apoptosis-related signaling molecules, and growth factors related with CTGF during differentiation, we performed immunoblotting analysis for TGF-β, β-catenin, JNK/p38, and AKT during the CTGF-induced differentiation of the hESCs. Based on several previous studies [13, 14, 19], we hypothesized that the expression of TGF-β and the canonical Wnt signaling pathway could be activated by CTGF. According to our hypothesis, first, we examined the relative expression value of CTGF, TGF-β, and β-catenin in the BM-MSCs, hESC-MSCs, and C-hESC-MSCs.

**Fig. 5:**
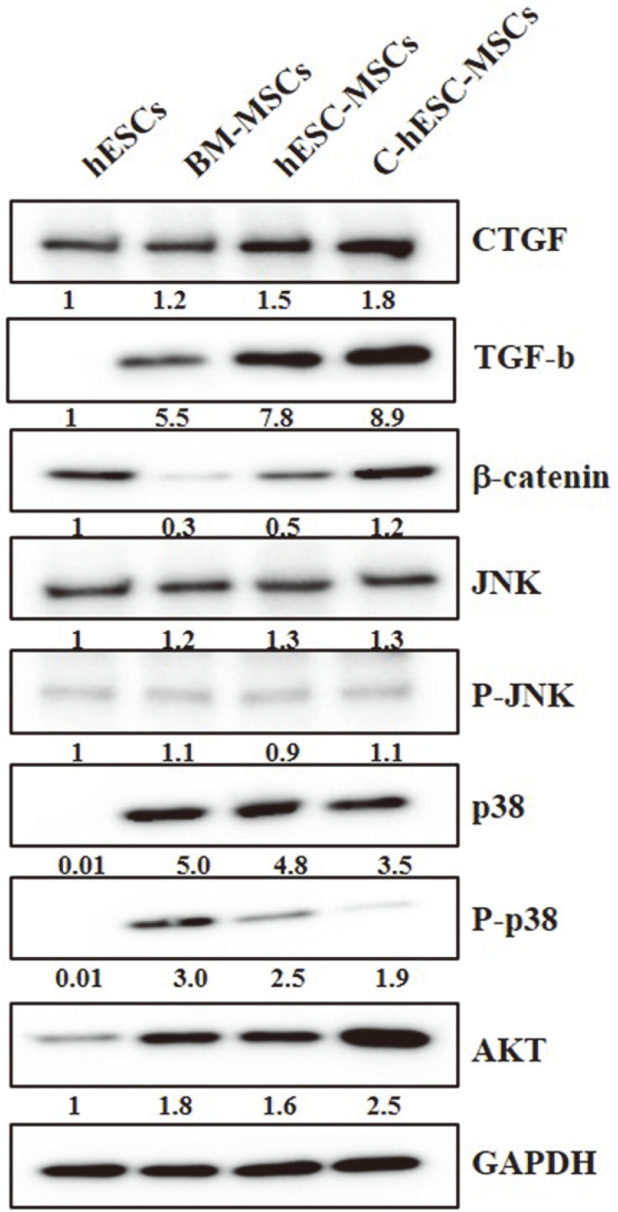
Verification of the cell signaling pathway-related molecules. Confirmation of the profibrotic growth factors (CTGF and TGF-β), Wnt/β-catenin signaling-related molecule (β-catenin), and cell survival/apoptosis-related signaling molecules, AKT and Jun amino-terminal kinases (JNK) (p-JNK)/p38 (p-p38), in the BM-MSCs, hESC-MSCs, and C-hESCMSCs (lane 1, hESCs; lane 2, BM-MSCs; lane 3, hESC-MSCs; lane 4, C-hESC-MSCs).

As shown in Fig. 5, as compared to the expression of the other molecules, the expression of TGF-β was the most up-regulated in the C-hESC-MSCs, along with the increased CTGF expression pattern, and the expression of β-catenin was also the most upregulated in the C-hESC-MSCs, similar to the TGF-β expression pattern. To determine the regulatory expression of cell survival/apoptosis-related signaling molecules in the CTGF-hESC-MSCs, we focused on the expression levels of the anti-apoptotic and pro-apoptotic molecules, including AKT and JNK (p-JNK)/p38 (p-p38) (Fig. 5).

Based on the western blotting analysis, we confirmed that the expression of p-JNK was almost similar in all three types of MSCs, but p-p38 was dramatically decreased in the C-hESC-MSCs, while AKT was markedly enhanced in the C-hESC-MSCs than in the BM-MSCs and hESC-MSCs (Fig. 5).

## Discussion

In the recent decade, several studies on stem cells, including embryonic and adult stem cells, have been increasing, owing to the regenerative potential in cell replacement therapy field for degenerative diseases, such as cardiovascular diseases, liver diseases and brain injury, resulting from their unique capacities of unlimited proliferation, plasticity, multipotency and pluripotency [2, 5, 20] In the present study, we successfully differentiated hESCs into MSCs and verified their characteristics as MSCs. We then further examined the molecular expression profile of C-hESC-MSCs using high-throughput proteomic analysis and identified differentially expressed proteins and their relationship in cell signaling pathway.

MSCs contribute to tissue repair in vivo as an attractive cell source, because they possess broad spectrum of lineage differentiation towards neuro-ectodermal (neurons, astrocytes, oligodendrocytes), mesodermal (adipocyte, chondrocyte, osteocyte) and endodermal (hepatocytes), also immuno-modulatory capacities. There are criteria for uniform characterization of MSCs such as plastic adherence, potential for differentiation into a variety of cell types, cell surface expression of CD105, CD73, CD90 and the absence of hematopoietic markers CD45, CD34, CD14 or CD11b, CD79α or CD19 and HLA-DR [5].

Results obtained from completed and on-going basic research and preclinical /clinical studies indicate great therapeutic potential of stem cell-based therapy in the treatment of degenerative, autoimmune and genetic disorders. However, clinical application of stem cells raises safety concerns, such as teratoma formation and activation of unknown signaling mechanisms after cell transplantation. That`s why there must need unraveling the molecular profile and underlying therapeutic mechanism in stem cell biology and therapy.

In addition, it must be preceded that establishment of therapeutic cellular source without limitation before stem cell application. The approach of in vitro differentiation of hESCs into MSCs may have huge potential for cell replacement therapy, because hESCs can be offered unlimited cellular source. And establishment of feeder-free culture and differentiation in chemically defined condition can be secure valuable therapeutic cellular source without xeno-contamination.

In this study, we obtained MSC population above mentioned chemically defined culture/differentiation condition from hESCs through CTGF treatment, without xeno-contamination. CTGF is a 38kDa extracellular matricellular protein that belongs to the CCN cysteine-rich family of proteins. Connective tissue growth factor is a multi-modular molecule that interacts with integrin receptors and several growth factors such as TGF-β, BMP, and insulin-like growth factor and allows networking between growth factors, the ECM, and cell surface receptors such as integrins. Also, CTGF modulates several cellular processes including cell adhesion, migration, proliferation, chemotaxis, apoptosis, ECM deposition, and angiogenesis under normal or pathological conditions. It has been documented that CTGF is aberrantly expressed in human cancer, is linked to the promotion or inhibition of the carcinogenic process, and is produced by several types of stromal cells, including tumor endothelial cells, vascular smooth muscle cells, and cancer-associated fibroblasts [11, 21].

It was also reported that CTGF participates in EMT by interacting with downstream proteins, inducing the expression of the integrin-linked protein kinase (ILK) protein and affecting the activity of bone morphogenic protein 7 precursor (BMP-7) and hepatocyte growth factor (HGF), and by activating the canonical Wnt signaling in vitro [13].

Proteome mapping serves as a starting point for developing a universal database for a given cell proteome and has proven to be extremely useful for analyzing complex protein expression patterns and their relationship between several kinds of signaling pathways. Using proteomics to investigate the processes that control the underlying molecular mechanisms of cells should provide valuable insights into how a cell contributes to recovery mechanisms in vivo and which cell types are the most optimal cellular sources for transplantation for a specific pathological condition. Also, applying proteomics to investigate the programs that control cell fate should provide valuable insights in understanding how the factors determine their different potential applications.

In this study, among the 3,000 total protein spots, 51 differentially expressed proteins varied significantly according to their cellular types by intensities of at least 1.5-fold in the hESC-MSCs and C-hESC-MSCs, respectively, relative to the BM-MSCs, and these proteins are listed in Table 1. A database search and functional exploration of these differentially expressed proteins revealed that these proteins have different roles according to their biological processes (Fig. 3C) and were divided into nine categories, including single-organism metabolic process (20%), negative regulation of metabolic process (12%), regulation of molecular function (13%), cell proliferation (10%), activation of immune response (5%), response to endogenous stimulus (9%), positive regulation of signaling (9%), regulation of biological quality (14%), and cell cycle proves (8%). According to their molecular function (Fig. 3D), the differentially expressed proteins were divided into 10 categories, including oxidoreductase activity (4%), lyase activity (3%), protein binding (27%), small molecule binding (11%), enzyme regulator activity (6%), heterocyclic compound binding (18%), macromolecular complex binding (7%), organic cyclic compound binding (18%), lipid binding (4%), and structural constituent of cytoskeleton (2%).

The 12 proteins that had the highest changes in expression were XRCC5, IGF2BP1, RBBP4, PCNA, NPM1, GNAI2, PHB2, LAMB1, LAMC1, ANXA3, PSME3, and PSMD5, and these were analyzed, described, and referred to the context of the study as the most promising target molecules that could be involved in the therapeutic mechanism of MSCs. Overall, the study aimed to provide valuable insight into the molecular functioning of in vitro C-hESC-MSCs.

XRCC5 is a single-stranded DNA-dependent, adenosine triphosphate (ATP)-dependent helicase, which has a role in chromosome translocation and double-strand break repair via nonhomologous end joining in mammalian cells. In previous studies, it was reported that XRCC5 promotes cancer cell growth (colon cancer and breast cancer), and the inhibition of XRCC5 using siRNA have been show to suppress cancer cell growth in vitro and, conversely, the overexpression of XRCC5 promoted cancer cell growth [22]. Moreover, XRCC5 plays a role in brain development, and cell population proliferation and performs an essential role in telomere maintenance in human cells [23]. The DNA damage response (DDR) is central for the maintenance of genome stability and includes transcriptional regulation, cell cycle arrest, DNA repair, and apoptosis when DNA breaks are not repaired. The DDR also triggers a broad post-transcriptional reprogramming, modulating the expression of genes involved in DNA repair, cell cycle control, and/or apoptosis. Thus, XRCC5 is important in the performance of MSCs functionality in vitro and in vivo [24]. In an miRNA study, it was reported that miR-623 suppressed cell proliferation, migration, and invasion through the downregulation of cyclin-dependent kinases, and the phosphatidylinositol-3-kinase (PI3K)/Akt and Wnt/β-catenin pathways were inhibited by targeting XRCC5 [23].

Insulin-like growth factor 2 mRNA-binding protein 1 is member of the IGF-II mRNA-binding protein (IMP) family and functions by binding to the 5' UTR of the insulin-like growth factor 2 (IGF2) mRNA and regulating IGF2 translation. Moreover, IGF2BP1 plays a key role in neurite outgrowth, growth cone guidance, neuronal cell migration, and neuronal stem cell population maintenance during neuronal development. Also, IGF2BP1 is an oncofetal mRNA-binding protein expressed in various cancers including leukemia, squamous cell carcinoma, and non-small cell lung cancer and in different stem cell types such as BM-MSCs, adipose tissue derived MSCs, and UCB-hematopoietic stem cells, and these expression patterns could correlate with their proliferation and tumorigenicity potential [25-27].

Histone binding protein 4, also known as chromatin-remodeling factor RBAP48 (RbAp48), is an evolutionarily conserved protein that has been involved in various biological processes such as promotion of cell proliferation and invasion ability [28]. Also, RBBP4 has essential roles in early mammalian embryogenesis, and loss of RBBP4 results in defective inner cell mass, severe apoptosis, hyperacetylated histones, and preimplantation lethality in mice [29]. RBBP4 activity is correlated with increased activity of the Wnt/β-catenin pathway [30].

Proliferating cell nuclear antigen is a DNA clamp that acts as a processivity factor for DNA polymerase in eukaryotic cells and is an essential protein that participates in a variety of processes of DNA metabolism, such as DNA replication, synthesis, chromatin organization, transcription, and repair [31]. In addition, PCNA acts as a cell proliferation marker in its application in toxicology and as a prognostic marker in human tumors [32].

Nucleophosmin 1, also known as B23, No38, or Numatrin, is a critical cellular protein found in the nuclei of proliferating cells, and the functions of NPM1 are also critical for cell growth and homeostasis, including the regulation of ribosome biogenesis, mRNA processing, chromatin remodeling, and stress response. Based on these functions, NPM1 is very important in sustaining genetic stability and DNA repair and us related with cancer development, such as in leukemia [33-36].

Guanine nucleotide-binding protein G(i) alpha-2 subunit is involved in a wide variety of signaling events mediated by the G-protein-coupled receptor (GPCR) as modulators or transducers in various transmembrane signaling systems and may play a role in cell cycle, cell division, and cell population proliferation [37, 38].

In in vitro experiments using many different types of tumors such as ovarian cancer cells, hepatocellular carcinoma, and tongue squamous cell carcinoma, gain-of-function mutations that led to the constant activation of GNAI2 have been identified in a number of cancers, which prompt the growth and survival of cancer cells [39]. On the other hand, the knockdown of GNAI2 via miRNA led to reduced proliferation, cell cycle arrest, apoptosis, and migration through the inactivation of the PI3K/AKT pathway because of AKT phosphorylation [38, 40]. In other studies, it has also been reported that GNAI2 enhances cell survival by activation of AKT and suppresses apoptosis by regulating B-cell lymphoma 2 (Bcl-2) expression [41].

Laminin is thought to mediate the attachment, migration, and organization of cells into tissues during embryonic development by interacting with other ECM components. Due to the various roles of laminin, they are a prerequisite for normal embryonic development [42]. Among the family of laminins, LAMB1 and LAMC1 are families of ECM glycoproteins and are the major non-collagenous structural constituents of basement membranes. Also, they are thought to be implicated in a wide variety of biological processes and pathological processes, including cell proliferation, adhesion, differentiation, migration, invasion, signaling, neurite outgrowth, metastasis, tissue repair, and organization of cells into tissues during embryonic development by interacting with other ECM components [43, 44]. In a siRNA study for LAMC1, the cell proliferation, migration, and invasion were depressed significantly after the downregulation of the LAMC1 expression [45], promoting cell death [46].

Prohibitin 2, a conserved multifunctional protein, is traditionally localized in the mitochondrial inner membrane and is essential for the maintenance of mitochondrial function, which include the plasma membrane-associated cell signaling functions, mitochondrial chaperone, and transcriptional co-regulator of transcription factors and sex steroid hormones in the nucleus [47, 48]. Knockdown of PHB2 in cancer cells induced the inhibition of cell proliferation through induction of cell cycle arrest and suppression of DNA synthesis. Meanwhile, the downregulation of PHB2 also induced apoptosis and promoted differentiation in fractions of rhabdomyosarcoma cells [49] and played a crucial role in the adhesion processes in the cell, thereby sustaining cancer cell propagation and survival [50]. Also, PHB2 participates in the homeostasis and differentiation of ES cells. PHB2 was highly expressed in undifferentiated mouse ES cells, and knockdown of PHB2 induced significant apoptosis in pluripotent ES cells, whereas the expression was decreased during the differentiation of the ES cells. Enhanced expression of PHB2 contributed to the proliferation of ES cells. However, enhanced expression of PHB2 strongly inhibited ES cell differentiation into neuronal and endodermal cells [51].

Annexin A3 is a calcium-dependent phospholipid-binding protein family and membrane-binding proteins that plays a role in the regulation of cellular growth and in signal transduction pathways such as membrane transport and other calmodulin-dependent activities on the membrane surface. Annexin A3 is involved in regulating inflammatory responses, cell differentiation, proliferation, apoptosis, migration, invasion, and interactions of cytoskeletal proteins [52]. The expression of ANXA3 is associated with multiple human diseases. For instance, annexin can mediate cell signaling pathways, cell movement, tumor invasion and metastasis, cell apoptosis, and drug resistance in the development and progression of tumors. As a member of the annexin family, ANXA3 plays an important role in tumorigenesis, cell proliferation, apoptosis, invasion, metastasis, and drug resistance. Annexin A3 could play an important role in the development, proliferation, migration, and metastasis of malignant tumors and/or tumor cell [53, 54].

Proteosome activator complex subunit 3, also referred to as REGγ, Ki antigen, and PA28γ, was first cloned in 1990 from a patient with systemic lupus erythematosus. The vital functions of PSME3 in the regulation of mitosis, immune response, and cell destiny determination, both at psychological and pathological status, have been revealed by numerous studies [55]. Aberrant overexpression of PSME3 has been revealed in a plethora of cancers, including pancreatic cancer, thyroid carcinoma, breast cancer, melanoma, oral cancer, non-small-cell lung cancer, and multiple myeloma [56]. Essentially, PSME3 serves as an oncogene, as is indicated by the existing studies, and promotes tumor apoptosis resistance, growth, and metastasis by modulating epithelial–mesenchymal transition, stem cell characteristics, and glycolysis in a Myc6-, Hippo-, or NF-kB13-associated manner. In conclusion, PCCs exert proliferative effects on PSCs through the PSME3-mediated AP-1/TGFB1 secretion pathway.

Proteosome 26S subunit non-ATPase 5 acts as a chaperone during the assembly of the 26S proteasome, specifically, of the base subcomplex of the PA700/19S regulatory complex. Mutations in genes encoding the non-ATPase subunits of the 19S regulator base of 26S proteasome complex have been associated with ophthalmological and vertebral defects in humans. Specifically, mutations in the *PSMD5* gene resulted in ocular coloboma and vertebral defects in zebrafish, suggesting an important role for PSMD5 during optic fissure closure and vertebral development [57]. The 26S proteasome is an enzymatic complex that degrades ubiquitinated proteins in eukaryotic cells. PSMD5 is one of a number of chaperones that are involved in the assembly of the proteasome. The chaperones dissociate before 26S proteasome formation is complete [58].

As shown in Figs. 4A and 4B, similar to that in the 2-DE analysis, the 12 specifically upregulated proteins also retained upregulated expression patterns in the C-hESC-MSCs when compared with those of the hESC-MSCs and BM-MSCs.

It is well known that CTGF may be regulated and most significantly upregulated by both the TGF-β superfamily, including BMPs, and Wnt3A, as a potential target of Wnt and BMP signaling [59]. The TGF-β and CTGF are profibrotic growth factors, downstream from nuclear translocation of β-catenin, that lead to increased fibrogenesis [19]. The Wnt signaling pathway contributes to cell proliferation, migration, mesodermal differentiation, and lineage commitment, including hematopoiesis, adipogenesis, osteogenesis, and so on. The CTGF stimulates phosphorylation of LRP6 and GSK-3b, resulting in accumulation and nuclear localization of β-catenin, TCF/LEF activity, and expression of Wnt targets and modulated Wnt signaling [13, 14]. Also, Wnt/β-catenin signaling is a pivotal regulator of MSCs and plays an important role in cell fate determination and mesodermal differentiation. It is well known that Wnt–β-catenin signaling regulates the expression of CTGF through the MAPK pathway and controls the fate of MSCs. Under the MAPK pathway, the ERK and AKT are typically thought to be antiapoptotic, whereas p38 and JNK are primarily pro-apoptotic [60].

In this study, we successfully induced hESC-MSCs via CTGF treatment and sought to determine the functional role of CTGF in MSC differentiation and cell signaling pathway.

We confirmed the proteomic analysis results using immunoblotting analysis and demonstrated that CTGF was upregulated, and the expression of TGF-β and β-catenin was also shown to be increased in the C-hESC-MSCs when compared with those of the BM-MSCs and hESC-MSCs (Fig. 5A). Based on the results, we could conclude that CTGF could activate the canonical Wnt signaling pathway in C-hESC-MSCs during differentiation, and it is expected to be able to contribute directly or indirectly to mesenchymal differentiation of hESCs more effectively. Our results elaborate the important role of CTGF as an activator of the canonical Wnt pathway in hESC differentiation into MSCs.

It has already been reported that Wnt/β-catenin signaling regulates the expression of CTGF through the MAPK pathway. Understanding the balance between Wnt/β-catenin signaling and CTGF is necessary for developing therapeutic alternatives [11]. There are at least three distinct MAPK signaling modules that mediate extracellular signals into the nucleus to turn on the responsive genes in mammalian cells. These include AKT, ERK, JNK, and p38 kinase. The MAPK signaling pathway is essential in regulating many cellular processes, including inflammation, cell stress response, cell differentiation, cell division, and cell proliferation, metabolism, motility, and apoptosis. The role of the MAPK pathway in cancer, immune disorders, and neurodegenerative diseases has been well recognized [61, 62].

Since its initial discovery as a proto-oncogene, the serine/threonine kinase Akt (also known as protein kinase B or PKB) has become a major focus of attention because of its critical role in regulating diverse cellular functions, including metabolism, growth, proliferation, survival, transcription, and protein synthesis.

Mitogen-activated protein kinase cascades are universal triple signaling pathways, Akt is known as a survival kinase and a main downstream target of the phosphoinositide 3-kinase (PI3K) [63]. Protein kinase B regulates cell growth through its effects on the TSC1/TSC2 complex and mTORC signaling. Moreover, Akt contributes to cell proliferation via phosphorylation of the CDK inhibitors p21 and p27, and Akt is a major mediator of cell survival through the direct inhibition of pro-apoptotic proteins like Bad or inhibition of pro-apoptotic signals generated by transcription factors like FoxO.

Jun amino-terminal kinases are members of the MAPK family and are activated by a variety of environmental stresses, inflammatory cytokines, growth factors, and GPCR agonists. The JNK kinase family consists of JNK1, JNK2, and JNK3. Jun amino-terminal kinases 1 and 2 are present in all tissues; however, JNK3 is only located in the brain, heart, and testes. JNK is linked to the transformation of oncogene and growth factor pathways. Irregularities in JNK activity have been linked to cancer, diabetes, inflammatory disorders, and neurodegenerative disorders [64, 65].

The kinase p38 is activated in response to a variety of extracellular environmental stresses, including osmotic shock, and inflammatory cytokines, lipopolysaccharides (LPS), anisomycin, UV light, and growth factors. The activation of p38 is mediated by several upstream kinases, including MAP kinase kinase 3 (MKK3), MAP kinase kinase 6 (MKK6), and MAP kinase kinase 4 (MKK4, also known as SEK1 and JNKK1). These kinases phosphorylate p38 at threonine 180 and tyrosine 182, resulting in p38 activation. Kinase p38 is linked to asthma, autoimmunity, and inflammation [62, 66].

In addition, based on the western blotting analysis (Fig. 5), we could confirm that the expression of p-JNK were almost similar in all three types of MSCs, but p-p38 were dramatically decreased in the C-hESC-MSCs, while AKT were markedly enhanced in the C-hESC-MSCs than in the BM-MSCs and hESC-MSCs.

The major and novel findings of our study include the development of the C-hESC-MSCs and activation of the canonical Wnt signaling pathway by CTGF treatment, which led to the activation of cell survival signaling including Akt and downregulation of JNK and p38 in the C-hESC-MSCs.

In this study, we have successfully directed the differentiation of hESCs into MSCs via CTGF treatment under fully chemically defined culture conditions, and C-hESC-MSCs showed broad MSC potential, illustrating their potential application in stem-cell-based regeneration therapy. The upregulated expression of cell proliferation-related molecules and signaling pathways in the proteomics analysis demonstrate the potential application of C-hESC-MSCs in stem cell-based therapy for the treatment of degenerative and autoimmune diseases. Although future studies should explore different endeavors, including treating genetic disorders and generating new stem cell-derived human tissues and biomaterials for use in pharmacy genomics and regenerative medicine, our results could widely contribute in the basic science and clinical application of stem cells.

## Figures and Tables

**Table 1:** Identification of differentially expressed proteins in BM-MSCs, hESC-MSCs and C-hESC-MSCs.

Spot No.	Accession No.*	Identified proteins	Seq. Cov (%)^†^	Matched peptide	pI	Mass (Da)	Mascot Score	Fold change		

								BM MSCs	hESC MSCs	C-hESC MSCs
1	IPI00182126	FK506-binding protein 9	18	13	4.91	63508	247	10	3	3
2	IPI00294380	Phosphoenolpyruvate carboxykinase [GTP], mitochondrial	37	26	7.56	71462	603	5	1	1
3	IPI00554786	Isoform 5 of Thioredoxin reductase 1, cytoplasmic	42	28	6.07	55484	754	15	4	3
4	IPI00789155	cDNA FLJ31776 fis, clone NT2RI2008141, highly similar to CALUMENIN	23.5	11	4.3	38143	295	20	5	5
5	IPI00220709	Isoform 2 of Tropomyosin beta chain	36.3	44	4.4	33028	1345	10	5	4
6	IPI00930609	Isoform 1 of Phosphoserine aminotransferase	37	20	7.56	40803	400	20	8	8
7	IPI00930609	Isoform 1 of Phosphoserine aminotransferase	15	5	7.56	40803	137	20	5	4
8	IPI00744692	Transaldolase	12	4	6.36	37690	146	20	7	6
9	IPI00005161	Actin-related protein 2/3 complex subunit 2	34	11	6.84	34428	164	30	25	10
10	IPI00303602	Axin interactor, dorsalization-associated protein	9	3	6.13	35175	82	20	1	1
11	IPI00217966	L-lactate dehydrogenase	17	6	8.61	40102	132	25	1	2
12	IPI00219616	Ribose-phosphate pyrophosphokinase 1	26	10	6.51	35334	195	30	5	25
13	IPI00295386	Carbonyl reductase [NADPH] 1	51	15	8.55	30646	402	50	1	1
14	IPI00010810	Electron transfer flavoprotein subunit alpha, mitochondrial	10	3	8.62	35406	44	20	2	2
15	IPI00793199	annexin IV	42	19	5.84	36294	672	20	5	5
16	IPI00479722	Proteasome activator complex subunit 1	24	6	5.78	28879	115	20	2	2
17	IPI00384051	Putative uncharacterized protein PSME2	51	15	6.05	28758	432	25	2	2
18	IPI00027681	Nicotinamide N-methyltransferase	24	7	5.56	30019	225	20	3	3
19	IPI00020906	Inositol monophosphatase	29	6	5.16	30575	152	30	1	1
20	IPI00645078	Ubiquitin-like modifier-activating enzyme 1	23	32	5.49	118876	655	1	20	20
21	IPI00220834	X-ray repair cross-complementing protein 5	30	22	5.55	83232	412	5	20	20
22	IPI00008524	Isoform 1 of Polyadenylate-binding protein 1	15	10	9.52	70858	162	2	25	25
23	IPI00008557	Insulin-like growth factor 2 mRNA-binding protein 1	17	10	9.26	63765	166	1	30	30
24	IPI00375441	Isoform 1 of Far upstream element-binding protein 1	12	10	7.18	67692	170	2	20	20
25	IPI00027834	Heterogeneous nuclear ribonucleoprotein L	13	8	8.46	64730	123	10	30	30
26	IPI00021808	Histidyl-tRNA synthetase, cytoplasmic	10	6	5.72	57954	105	2	30	30
27	IPI00008475	Hydroxymethylglutaryl-CoA synthase, cytoplasmic	4	3	5.22	57837	53	1	22	22
28	IPI00328319	Histone-binding protein RBBP4	10	5	4.74	47916	112	5	20	20
29	IPI00022694	Isoform Rpn10A of 26S proteasome non-ATPase regulatory subunit 4	5	2	4.68	40943	77	7	20	20
30	IPI00418471	Vimentin	53	36	5.06	53677	942	10	20	20
31	IPI00418471	Vimentin	53	11	5.06	53677	367	1	20	17
32	IPI00418471	Vimentin	73	110	5.06	53677	2371	1	20	15
33	IPI00549248	Isoform 1 of Nucleophosmin	34	29	4.64	32729	837	5	30	25
34	IPI00021700	Proliferating cell nuclear antigen	19	8	4.57	29098	92	1	20	17
35	IPI00748145	Isoform 1 of Guanine nucleotide-binding protein G(i), alpha-2 subunit	13	5	5.34	41005	109	5	25	26
36	IPI00008552	Glutaredoxin-3	17	5	5.31	37698	67	4	25	25
37	IPI00027341	Macrophage-capping protein	7	3	5.88	38784	120	3	30	25
38	IPI00217223	Multifunctional protein ADE2	28	19	7.44	50402	274	5	50	40
39	IPI00455315	Annexin A2	53	22	7.57	38812	528	20	25	5
40	IPI00027252	Prohibitin-2	35	13	9.83	33276	196	2	30	20
41	IPI00011253	40S ribosomal protein S3	73	37	9.68	26845	845	5	40	35
42	IPI00024095	Annexin A3	43	18	5.63	36527	438	2	20	40
43	IPI00030243	Isoform 1 of Proteasome activator complex subunit 3	30.7	7	5.6	29604	198	5	10	20
44	IPI00643746	High-mobility group box 1	41	4	9.91	11461	36	5	30	2
45	IPI00018755	High mobility group protein 1-like 10	31	8	6.99	24377	128	2	30	2
46	IPI00013976	Laminin subunit beta-1	8	17	4.84	205303	233	0	1	20
47	IPI00298281	Laminin subunit gamma-1	17	39	5.01	183289	1127	0	1	21
48	IPI00002134	26S proteasome non-ATPase regulatory subunit 5	34	21	5.35	56566	559	5	10	30
49	IPI00002134	26S proteasome non-ATPase regulatory subunit 5	19	8	5.35	56566	111	1	3	30
50	IPI00013847	Cytochrome b-c1 complex subunit 1, mitochondrial	23	16	5.94	53308	341	5	10	2
51	IPI00017596	Microtubule-associated protein RP/EB family member 1	41	11	5.02	30154	326	5	10	15

*IPI (International Protein Index) accession numbers.

^†^The percentage of matched sequence in the total protein sequence (the percentage of the database protein sequence covered by matching peptides).
